# Demographic and Pathological Features of 430 Cases of Lymphoma from a Tertiary Hospital in Seremban, Malaysia (2015–2022)

**DOI:** 10.21315/mjms-03-2025-214

**Published:** 2025-10-31

**Authors:** Kean Ghee Lim, Muhammad Nabil Nasarudin, Hui Yang Catherine Khaw, Jing Yee Lim, Nuha Sabrina Nordin, Aishath Ana Ibrahim Hameed, Noor Hasni Shamsudin, Ismail Abdul Sattar Burud, Suneet Sood

**Affiliations:** 1 Department of Surgery, IMU University, Clinical Campus, Seremban, Negeri Sembilan, Malaysia; 2 Department of Pathology, Hospital Tuanku Ja’afar, Seremban, Negeri Sembilan, Malaysia

**Keywords:** lymphoma, Hodgkin, follicular, large B-cell

## Abstract

**Background:**

Lymphoma, including both Hodgkin and non-Hodgkin types, is the fourth most common cancer in Malaysia. Few large studies have examined the frequencies and patterns of occurrence. This study describes the epidemiology of lymphoma in Malaysia based on cases from a public hospital, and compares demographic characteristics, distribution, subtypes, and anatomical sites.

**Methods:**

This retrospective study was conducted at Hospital Tuanku Ja’afar, Seremban, Malaysia. Admitted patients who had received medical or surgical treatment for lymphoma between 2015 and 2022 were reviewed.

**Results:**

A total of 430 cases were recorded. The distribution between extranodal (52.1%) and nodal (46.0%) lymphoma was nearly equal. Hodgkin lymphoma (HL) accounted for 16.3% of cases, with a mean age at presentation of 37 years, compared with 55.7 years for non-Hodgkin lymphoma (NHL). Diffuse large B-cell lymphoma (DLBCL) was the most common NHL subtype, representing 44.2% of all lymphoma cases. Among patients with staging information, 63% were diagnosed at stage IV. Males comprised 61% of the population. The predominant ethnic group was Malay (70.2%). The largest age group was 60 to 69 years (32.2%). Follicular lymphoma occurred less frequently than in Western and other Asian countries but was more common among ethnic Indians in Malaysia. Primary mediastinal large B-cell lymphoma, like other large B-cell lymphomas, was more frequent among Malays. Ethnic Indians also showed higher rates of HL (30.7%) and lower rates of DLBCL (25.6%).

**Conclusion:**

This study highlights many similarities in the distribution of lymphoma in Malaysia compared with patterns observed in the region and globally.

## Introduction

Lymphoid malignancies are increasing worldwide, including in Malaysia (1, 2). Mature lymphomas are haematological malignancies arising as discrete tissue masses and are broadly classified into Hodgkin lymphoma (HL) and non-Hodgkin lymphoma (NHL). Globally, NHL ranks 10th in incidence and 11th in mortality, while HL ranks 26th and 28th, respectively (3, 4). In Malaysia, NHL ranks 6th for incidence and 7th for mortality, while HL ranks 23rd and 27th. Together, HL and NHL account for 2,402 cases and 1,382 deaths annually (5).

HL has distinct clinical features and treatment outcomes, while NHL encompasses over 80 subtypes. The WHO classification, introduced in 2001 and updated most recently in 2022, incorporates morphology, immunohistochemistry, and molecular genetics (6, 7).

Malaysia, with a population of 34.1 million, is multiethnic, comprising Malay, Chinese, Indian, and Bumiputera groups, and migrants (8). Healthcare is two-tiered: 20% to 30% of patients use private care, but prolonged or costly treatments often compel them to return to the public system (9, 10). Hospital Tuanku Ja’afar, Seremban (HTJS), the largest public hospital in Negeri Sembilan, serves a population of 1.24 million, including 6% of non-citizens (8).

The largest Malaysian series of lymphoma cases to date (11) reported 210 cases from a private hospital. Eight earlier series between 1980 and 2006 reported 70 to 149 cases each (12), and one series included 24 children (13). Reviews have summarised case reports and outcomes (12, 14, 15), but no large public hospital series using the WHO classification has analysed subtype distribution. This study addresses that gap.

## Methods

We conducted a retrospective study of patients diagnosed with lymphoma at HTJS between 2015 and 2022. Diagnosis was based on histology and immunohistochemistry. A second opinion of a lymphoreticular histopathologist was routinely sought for difficult cases. Patients diagnosed prior to 2015 and those with concurrent malignancies, leukaemia, or immature lymphomas were excluded. We obtained approval from the Medical Research and Ethics Committee, Ministry of Health Malaysia [NMRR ID-23-02984-GWV (IIR)], as well as the Hospital Director of HTJS, to access the medical records of HTJS.

We accessed the reports of the HTJS pathology department for all cases of mature lymphomas using the WHO classification. This was cross-checked with the department’s audit of patients on the National Cancer Registry. We extracted information regarding their histological types of lymphoma and the site of occurrence. The sites were classified according to the organ in which they were found. Where lymph nodes were involved, the location was noted when possible. Lymphoma found in the mediastinum was classed as extranodal unless specifically stated to be of a lymph node. Lymphoma from trephine biopsies of bone was considered metastatic disease, but considered primary from the bone when there was evidence for it. Patients who had other concurrent malignancies and patients with leukaemia and immature lymphomas were excluded.

Social and demographic features of patients were obtained from the medical records archives stored in HTJS. We obtained data such as their age, gender, race, type of lymphoma, year of lymphoma diagnosis and treatment received.

Confidentiality of the data collection process was ensured. The data sheet, which contained information from data collection, was protected with a password and did not contain any identifiers, such as the patient’s name, identification number, or any traceable codes.

Statistical analyses were performed using SPSS V29.0. Categorical variables were assessed with chi-square tests, and logistic regression was used to adjust for covariates. Artificial intelligence has not been employed to assist the data collection, analysis, nor writing of this paper.

## Results

### Sites of Lymphoma

Of 430 cases, 46.0% were nodal and 52.1% were extranodal (Table 1). The remaining 1.9% were unspecified. The commonest sites for lymph nodes were the cervical region (85, 42.7%), inguinal (35, 17.6%), supraclavicular (26, 13.1%) and axilla (16, 8.0%). Thirty-seven (18.6%) more were in various other sites and in 23 the site was not determined. The commonest extranodal lymphoma sites were the upper airway/pharynx, including 40 (17.9%) in the tonsils, and 35 (15.6%) in the nose, the nasopharynx, including the Fossa of Rosenmuller, the palate and tongue. There were 45 (20.1%) in the gastrointestinal tract, including 21 in the stomach, 12 in the colon and nine in small bowel, two in the rectum and one in the anus. The rest occurred in numerous other sites, notably 13 (5.8%) in the spleen, 12 (5.4%) in the brain and 11 (4.9%) in the skin. Fifty-two lymphoma cases had samples from bone or bone marrow, which were all assigned to other sites except eight, which are labelled unknown but considered stage IV as they were trephine bone samples. Three cases were considered primary in the bone.

### Subtypes

Lymphomas were grouped into 15 subtypes, namely diffuse large B-cell lymphoma (DLBCL), high grade B-cell, large B-cell [including primary mediastinal (PMBL) (10) and plasmablastic (1)], marginal zone (12) [including nodal marginal zone (4) and mucosa-associated lymphoid tissue (MALT) (2)], follicular, Burkitt, small lymphocytic, mantle cell, low grade B-lymphoma [including B lymphoproliferative disorder (2) and lymphoplasmacytic (1)], B-cell not-otherwise specified (NOS), Hodgkin, NK/T-cell, T-cell, anaplastic large cell lymphoma (ALCL) (3) and angioimmunoblastic T-cell lymphoma (ATL) (3) and lymphoma not-otherwise-specified (NOS), with counts for those grouped together given next to the name. Combining these groups did not significantly change the demographic distribution of the enlarged group.

NHL comprised 83% of cases. DLBCL was the most common subtype (44.2%), followed by HL (16.3%) (Table 1). DLBCL and its variants are largely extranodal; the subtype with highest extranodal occurrence was Burkitt lymphoma. Follicular lymphoma was rare (3.0%), but more common in ethnic Indians (Table 2). T-cell and NK/T-cell lymphomas were predominantly extranodal. PMBL accounted for 10 cases, mostly among Malays. The small/low grade B-cell variants tend to be nodal, except for marginal zone lymphoma. HL accounted for 16.3% and chiefly occurs in lymph nodes. T-cell and NK/T-cells lymphomas are mainly extranodal (74.1%, 23/31).

### Age

The median age was 58 years (range 7–89) (Table 1). HL presented at a mean age of 37 years versus 55.7 years for NHL (*P* < 0.001). The 60 to 69 years age group formed nearly one-third of cases. PMBL presented in younger patients (mean 23.8 years). Indolent subtypes (marginal zone, mantle cell) were confined to those over 50 years (Table 3).

### Gender

Males accounted for 61% of cases (Table 1). The distribution of nodal versus extranodal disease was similar between sexes. Females were more frequent among large B-cell lymphoma patients. However, 10 of these were PMBL, comprising six men and four women. The remaining nine large B-cell were two men and seven women. Mantle cell, ALCL, and ATL occurred exclusively in men (Table 2).

### Ethnicity

Binary logistic regression showed Chinese patients were more likely to have extranodal disease (OR 2.85, 95% CI: 1.62, 4.99) (Table 1). Non-Malays have disproportionately larger numbers of small/low grade B-cell lymphomas (28%) as compared to Malays (15%) (χ^2^ = 7.47, *P* = 0.006). Indians account for 9.1% of all lymphoma cases but are under-represented among the diffuse large B-cell variants (10/39 vs. 178/386) (χ^2^ = 6.0, *P* = 0.02) and overrepresented particularly in follicular lymphoma (4/39) compared to Malays and Chinese (9/386) (χ^2^ = 5.1, *P* = 0.02 with Yates correction). Indians were overrepresented in HL (12/39 vs. 57/386) (χ^2^ = 6.7, *P* = 0.01) (Table 2). Malays were overrepresented overall and accounted for eight of nine PMBL cases.

### Stage

Stage was recorded for 38.4% of patients (Table 1). Of these, 63% were stage IV at diagnosis. HL was more likely to be diagnosed at earlier stages (29% stages I–II). Among DLBCL patients, 54.4% were stage IV. There were no clear differences in the site of disease, gender, ethnic group or age group for the stage of disease. Missing data limited staging analysis. Burkitt’s lymphoma (54.5%) and follicular lymphoma (53.8%) were most likely to be staged.

HL was staged in 47.1% and was the lymphoma most likely to be in the early stage (29% stages I and II). In the largest subgroup, DLBCL, 68 (35.8%) were staged, 54.4% in stage IV, 13.2% in stage III, 27.9% in stage II and 4.4% in stage I.

## Discussion

This study represents the largest public hospital series of lymphomas in Malaysia. The near-equal nodal and extranodal distributions reflect Asian patterns but contrast with Western series (16).

### Subtypes

DLBCL predominance (44%) aligns with other Malaysian and Asian reports (11, 17, 18, 19) but exceeds Western rates (25% to 35%) (2, 20). HL (16.3%) matched global estimates (10% to 15%) (3, 4). The significantly different age group and ethnic distributions of HL also mark it out as a different disease from the rest of lymphomas. Follicular lymphoma was rare in our series, even compared with other Malaysian studies (11, 17, 18), Asian studies (2, 19) and much lower than in the Western world (2, 20) except among Indians, consistent with data from India (21).

Large B-cell lymphoma is an umbrella group noted in Table 13.3 in the WHO classification (6) which groups 13 lymphoma subtypes. Ten of our cases of large B-cell lymphoma are PMBL, which are usually more common in women and young adults (6). We have however, more men than women, but a large majority of Malays.

All four cases of NK/T lymphoma were nasal/nasopharynx in origin, consistent with the pattern seen in Asia (22).

### Age and Gender Distribution

The median age (58 years) was slightly higher than that reported for Salam’s private hospital cohort (50 years) (11). Male predominance (61%) matched global trends. Indolent lymphomas, namely marginal zone and mantle cell, that form 4.2% and 2.1% of all lymphoma respectively, appeared mainly in older patients.

We found that males made up 61% of the patients, reflecting global data. Mantle cell lymphoma occurs more frequently in males, but the distribution in this series, of only nine cases, all in men and none in women, is notable.

### Ethnicity

Malays are overrepresented in this series, and the National Cancer Registry records a higher incidence of lymphoma among Malays (10), which differs with the report from a private hospital, which had only 27% Malays (11). Similar to that study, Indians in our series have more HL and follicular lymphoma (11), as seen in New Delhi, India, where HL accounted for 30.4% of all lymphoma and follicular lymphoma for 7.5% (21). This pattern of lymphoma among Indians who have lived in Malaysia all their lives suggest that genetic factors rather than the environment drive HL and follicular lymphoma.

### Strengths and Limitations

The main strengths of this study are the large cohort size, multi-source verification, and the application of the WHO classification, which enable reliable subtype analysis. As a retrospective single-centre study, the limitations include missing data (especially for staging) and a lack of national representativeness, which limit generalisability.

## Conclusion

DLBCL is the most common lymphoma in Malaysia. It occurs more frequently than in Western countries. HL occurs in younger patients and shows ethnic variation. Follicular lymphoma is uncommon overall but relatively higher among Indians. PMBL occurs mainly in young Malays, without female predominance. These findings highlight important ethnic and regional patterns in lymphoma distribution.

## Figures and Tables

**Table 1:** Distribution of nodal and extranodal lymphomas among patients in Hospital Tuanku Ja’afar, Seremban (2015–2022)

Characteristic	Nodal *n* (%)	Extranodal *n* (%)	Undetermined *n* (%)	Total	

				*n* (%)	(95% CI)
	198 (46.0)	224 (52.1)	8 (1.9)	430	

**Sex**					
Male	121 (46.4)	134 (51.3)	6 (2.3)	261 (61.0)	(56, 65)
Female	77 (45.6)	90 (53.3)	2 (1.2)	169 (39.0)	(35, 44)

**Race**					
Malays	147 (48.8)	150 (49.8)	4 (1.3)	301 (70.0)	(74, 66)
Chinese	28 (32.9)	53 (62.4)	4 (4.7)	85 (19.8)	(16, 24)
Indians	22 (56.4)	17 (43.6)	0	39 (9.1)	(6, 12)
Others	1 (20)	4 (80)	0	5 (1.2)	

**Age**					
< 20	8 (44.4)	9 (50)	1 (5.6)	18 (4.2)	
20 to 29	27(56.3)	20 (41.7)	1 (2.1)	48 (11.2)	
30 to 39	30 (65.2)	15 (32.6)	1 (2.2)	45 (10.7)	
40 to 49	24 (53.3)	21 (46.7)	0	45 (10.5)	
50 to 59	26 (36.6)	45 (63.4)	0	71 (16.5)	
60 to 69	61 (43.9)	75 (54.0)	3 (2.2)	139 (32.3)	
> 70	22 (46.4)	39 (61.9)	2 (3.2)	63 (14.7)	

**Lymphoma type**					
Diffuse large B-cell variants				256 (59.5)	(55, 64)
DLBCL	71 (37.49)	117 (61.6)	2 (1.1)	190 (44.2)	
High grade B-cell	10 (27.8)	26 (72.2)	0	36 (8.4)	
Large B-cell	4 (21.1)	15 (78.9)	0	19 (4.4)	
Burkitt	2 (18.2)	9 (81.8)	0	11 (2.6)	
Small/low grade B-cell variants				57 (13.2)	(3, 16)
Marginal zone	4 (22.2)	14 (77.8)	0	18 (4.2)	
Follicular	10 (76.9)	3 (23.1)	0	13 (3.0)	
Small lymphocytic	9 (90)	1 (10)	0	10 (2.3)	
Mantle cell	7 (77.8)	1 (11.1)	1 (11.1)	9 (2.1)	
Low grade B-cell	1 (14.3)	4 (57.1)	2 (28.6)	7 (1.6)	
B-cell NOS	5 (71.4)	1 (14.3)	1 (14.3)	7 (1.6)	
Hodgkin	63 (90)	6 (8.6)	1(1.4)	70 (16.3)	
T-cell	8 (29.6)	19 (70.4)	0	27 (6.3)	
NK/T-cell	0	4 (100)	0	4 (0.9)	
ALCL + ATL	2 (33.3)	3 (50)	1 (33.3)	6 (1.4)	
Lymphoma NOS	2 (66.7)	1 (33.3)	0	3 (0.7)	

**Stage**					
I	4 (2.0)	3 (1.3)	4 (50)		
II	11 (5.5)	23 (10.3)			
III	11 (5.5)	9 (4.0)			
IV	46 (23.1)	54 (24.2)	4 (50)		
Undetermined	127 (63.8)	134 (60.1)			

CI = confidence interval; DLBCL = diffuse large B-cell lymphoma; NOS = not-otherwise specified; ALCL = anaplastic large cell lymphoma; ATL = adult T-cell lymphoma

**Table 2:** Gender and ethnic distribution of subtypes of lymphoma in Hospital Tuanku Ja’afar, Seremban (2015–2022)

Characteristic	Female *n* (%)	Male *n* (%)	Malay *n* (%)	Chinese *n* (%)	Indian *n* (%)	Others *n* (%)
**Diffuse large B-cell variants**						
DLBCL	86 (45.3)	104 (54.7)	140 (73.7)	38 (20)	10 (5.3)	2 (1.1)
High grade B-cell	17 (47.2)	19 (52.8)	27 (75)	7 (19.4)	2 (5.6)	0
Large B-cell	11 (57.9)	8 (42.1)	17 (89.4)	1 (5.3)	1 (5.3)	0
Burkitt	5 (45.5)	6 (54.7)	8 (72.7)	2 (18.2)	0	1 (9.1)
**Small/low grade B-cell variants**						
Marginal zone	4 (22.2)	14 (77.8)	10 (55.6)	7 (38.95)	1 (5.6)	0
Follicular	4 (30.8)	9 (69.2)	9 (69.2)	0	4 (30.8)	0
Small lymphocytic	3 (30.0)	7 (70)	5 (50)	5 (50)	0	0
Mantle cell	0	9 (100)	5 (55.6)	3 (33.3)	1 (11.1)	0
Low grade B-cell	2 (28.6)	5 (71.4)	4 (57.1)	2 (28.6)	1 (14.3)	0
**Other B-cell**						
B-cell NOS	1 (14.3)	6 (85.7)	4 (57.1)	2 (28.6)	1 (14.3)	0
Hodgkin	27 (38.6)	43 (61.4)	48 (68.6)	9 (12.9)	12(17.1)	1 (1.4)
**Non-B-cell**						
T-cell	6 (22.2)	21 (77.8)	17 (63)	6 (22.2)	4 (14.8)	0
NK/T-cell	2 (50)	2 (50)	1 (25)	2 (50)	0	1 (25)
ALCL + ATL	0	6 (100)	3 (50)	1 (16.7)	2 (33.3)	0
Lymphoma NOS	1 (33.3)	2 (66.7)	0	0	3 (100)	1 (11.1)

DLBCL = diffuse large B-cell lymphoma; NOS = not-otherwise specified; ALCL = anaplastic large cell lymphoma; ATL = adult T-cell lymphoma

**Table 3:** Age group distribution of subtypes of lymphoma in Hospital Tuanku Ja’afar, Seremban (2015–2022)

Age group (years)	< 20 *n* (%)	20 t0 29 *n* (%)	30 to 39 *n* (%)	40 to 49 *n* (%)	50 to 59 *n* (%)	60 to 69 *n* (%)	> 70 *n* (%)
**Diffuse large B-cell variants**							
DLBCL	5 (2.6)	9 (4.7)	13 (6.8)	22 (11.6)	38 (20.0)	68 (35.8)	35 (18.4)
High grade B-cell	1 (2.8)	2 (5.6)	4 (11.1)	2 (5.6)	4 (11.1)	12 (33.3)	11 (30.6)
Large B-cell	2 (10.5)	6 (31.6)	5 (26.3)	0	3 (15.8)	3 (15.8)	0
Burkitt	2 (18.2)	0	1 (9.1)	3 (27.3)	1 (9.1)	4 (36.4)	0
**Small/low grade B-cell variants**							
Marginal zone	0	0	0	0	1 (5.6)	14 (77.8)	3 (16.7)
Follicular	0	0	1 (7.7)	3 (23.1)	3 (23.1)	5 (38.5)	1 (7.7)
Small lymphocytic	1 (10)	1 (10)	2 (20)	0	1 (10)	2 (20)	3 (30)
Mantle cell	0	0	0	1 (11.1)	0	7 (77.8)	1 (11.1)
Low grade B	0	0	0	1 (14.3)	3 (42.9)	2 (28.6)	1 (14.3)
**Other B-cell**							
B-cell NOS	0	1 (14.3)	0	2 (28.6)	1 (14.3)	1 (14.3)	2 (28.6)
Hodgkin	7 (10)	23 (32.9)	18 (25.7)	5 (7.1)	8 (11.4)	7 (10)	2 (2.9)
Non-B-Cell							
T-cell	0	4 (14.8)	1 (3.1)	4 (14.8)	5 (18.5)	10 (37)	3 (11.1)
NK-/T-cell	0	1 (25)	0	1 (25)	0	1 (25)	0
ALCL+ATL	0	1 (16.7)	1 (16.7)	0	3 (50)	1 (16.7)	0
Lymphoma NOS	0	0	0	1 (33.3)	0	2 (66.7)	0

DLBCL = diffuse large B-cell lymphoma; NOS = not-otherwise specified; ALCL = anaplastic large cell lymphoma; ATL = adult T-cell lymphoma
